# 

**Published:** 1884-11

**Authors:** 


ORIGINAL COMMUNICATIONS:

The Herbst Method of Filling Teeth. C. F. W. Bodecker............ 597
A Consideration of the Comparative Merits of Watts' Crystal Gold among Filling Materials. J. F. P. Hodson........................ 607
Clinical Observations and Laboratory Experiments. C. M. Wright.... 613
The Bromides in Dentistry. R. M. Sanger ........................ 617
Higher Dental Attainments. J. G. Palmer........................... 621

REPORTS OF SOCIETY MEETINGS:

American Medical Association........................................ 623
American Dental Association ............ ........................ 626
New Jersey State Dental Society ................................. 631
Pennsylvania State Dental Society ....................... ......... 636
American Dental Society of Europe.............................. 641

EDITORIAL:

An Editor's Special............................................... 646
A Gross Misapprehension...................... ................... 647
Deceased.................................... ................... 648
The Missing Number. .......... .... ............................ 648
The Author.......................................................... 648
Our Book Table....................................... .. ... 649

CURRENT NEWS AND OPINION:

New Local Anæsthetic ................................ ........ 651
Mouth Mirrors....................................................... 651
Askings and Answers ... ........................................ 652
Please Remit.......................................................... 652
Assistant Needed.................................................... 652
Obituary ................................................................. 652

